# DNA metabarcoding assessment of Neotropical ichthyoplankton communities is marker‐dependent

**DOI:** 10.1002/ece3.10649

**Published:** 2023-10-20

**Authors:** Daniel Fonseca Teixeira, Heron Oliveira Hilário, Gilmar Bastos Santos, Daniel Cardoso Carvalho

**Affiliations:** ^1^ Post‐Graduate Program in Vertebrate Biology Pontifical Catholic University of Minas Gerais, PUC Minas Belo Horizonte Brazil; ^2^ Post‐Graduate Program in Genetics Federal University of Minas Gerais Belo Horizonte Brazil

**Keywords:** 12S rRNA, COI, high‐throughput DNA sequencing, molecular taxonomy

## Abstract

The study of ichthyoplankton is paramount to understanding fish assemblages' reproductive dynamics. DNA metabarcoding has been applied as a rapid, cost‐effective, and accurate taxonomy tool, allowing the identification of multiple individuals simultaneously. However, there remain significant challenges when using DNA metabarcoding, such as molecular marker choice according to the taxonomic resolution and length of the fragment to be sequenced, primer bias, incomplete reference databases, and qualitative inference incongruences. Here, 30 ichthyoplankton pools collected from a Neotropical river were identified at a molecular level using DNA metabarcoding to compare the resolution, sensibility, specificity, and relative read abundance (RRA) recovery of three molecular markers: the standard COI fragment (650 pb, with each end analyzed individually) and two short 12S rRNA genes markers (≅200 bp – NeoFish and MiFish markers). The combined use of the three markers increased the genera detection rates by 25%–87.5%, allowing an increased taxonomic coverage and robust taxonomic identification of complex Neotropical ichthyoplankton communities. RRA is marker‐dependent, indicating caution is still needed while inferring species abundance based on DNA metabarcoding data when using PCR‐dependent protocols.

## Graphical abstract

DNA metabarcoding has been applied as a rapid, cost‐effective, and accurate taxonomy tool, allowing the identification of multiple individuals simultaneously. The combined use of the three markers increased the genera detection rates by 25%–87.5%, allowing an increased taxonomic coverage and robust taxonomic identification of complex Neotropical ichthyoplankton communities. RRA is marker‐dependent, indicating caution is still needed while inferring species abundance based on DNA metabarcoding data when using PCR‐dependent protocols.

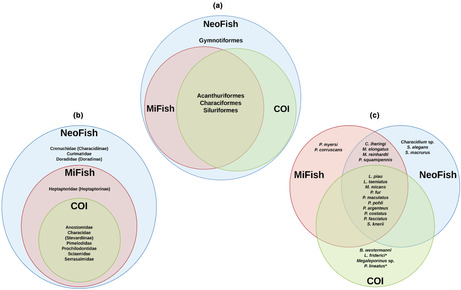

## 1 INTRODUCTION

The study of ichthyoplankton composition, abundance, and distribution is pivotal for understanding the reproductive dynamics of local fish assemblages (Mariac et al., 2018). The analysis of these parameters allows the identification of spawning sites, nursery areas where recruitment occurs, migration routes, temporal and spatial pattern variations, and differences in the reproduction patterns of migratory and nonmigratory fish (Baumgartner et al., 2004; Bialetzki et al., 2005; Reynalte‐Tataje et al., 2012). This information is instrumental in elucidating the influence of anthropogenic environmental alterations on fish reproduction and in the definition of effective management actions for species conservation and, consequently, fishing stock maintenance (da Silva et al., 2015; Silva et al., 2017).

Traditionally, ichthyoplankton taxonomy has applied the regressive development sequence technique, based on the morphological comparison of younger larvae with previously identified juveniles (Ahlstrom & Moser, 1976; Nakatani et al., 2001). However, due to the absence of morphological diagnostic characters during the egg stage, some authors exclude them from the studies and resort to identifying exclusively larvae, which in the initial stages is also a difficult task (Baumgartner et al., 2008; Reynalte‐Tataje et al., 2012). Moreover, the accuracy of the traditional morphological identification can diverge between taxonomists and laboratories, according to their experience and specialty (Ko et al., 2013). These limitations can compromise surveying essential information to conserve the areas of interest (Nobile et al., 2019).

Studies have employed molecular techniques to strengthen the precision and reliability of ichthyoplankton taxonomy. Comparative investigations have demonstrated that molecular taxonomy using DNA barcoding is more efficient than traditional morphological taxonomy, identifying the eggs and larvae to lower taxonomic levels and correcting erroneous morphological identifications (Becker et al., 2015; Ko et al., 2013). Using DNA barcoding, Frantine‐Silva et al. (2015) identified over 99% of 536 ichthyoplankton samples at species levels, including eggs, which accounted for 30% of the observed species richness. Morphologically, Becker et al. (2015) identified eggs only as migratory or nonmigratory, when possible, while DNA barcoding allowed the identification of eggs (plus damaged larvae) to species level, and highlighted imprecisions in the morphological taxonomy even with such coarser analysis. Nonetheless, despite its great taxonomic precision, DNA barcoding relies on individual processing and sequencing of each organism and can become expensive and laborious for large scale inventories (Taberlet et al., 2012; Yu et al., 2012), such as ichthyoplankton studies (Mariac et al., 2018; Nobile et al., 2019).

The DNA metabarcoding approach, using High‐Throughput Sequencing (HTS), has gained prominence for its ability to allow massive biodiversity access and transform ecology (Yu et al., 2012). The method combines DNA barcode‐based taxonomy with HTS to simultaneously identify hundreds to thousands of organisms. DNA metabarcoding analyses are economical, quick, broad, minimally dependent on taxonomic expertise, and its data remain available for further verification (Taberlet et al., 2012; Yu et al., 2012). This approach has allowed the reconstruction of ancestral communities (Jørgensen et al., 2012), biodiversity monitoring (Andersen et al., 2012), and detection of larger operational taxonomic units in a fraction of the time spent in conventional studies based on morphology and DNA barcoding (Fonseca et al., 2010). This approach has also shown high efficiency in ecological ichthyoplankton studies, allowing precise and reliable identification of fish egg and larva bulk samples (Kimmerling et al., 2018; Mariac et al., 2018).

Different from environmental samples (for example, soil and water), in which genetic material is often degraded, bulk samples usually provide genomic DNA of better quality, allowing the amplification of markers with longer sequences (Taberlet et al., 2012). However, the HTS platforms accessible to most research laboratories have limited sequencing lengths of up to 600 base pairs (bp). This hampers the usage of the full COI DNA barcoding, since its primers amplified a 650 bp fragment of the mitochondrial cytochrome c oxidase subunit I (COI) gene commonly used for fish (Ward, 2009). Additionally, the variability in COI sequences hinders the design of internal minibarcode primers, taking some researchers to pass this gene over in favor of more conserved ones for metabarcoding (Collins et al., 2019; Deagle et al., 2014). Among these conserved genes, mitochondrial 12S rRNA has been highlighted as a good alternative for fish metabarcoding (Milan et al., 2020; Miya et al., 2020; Sales et al., 2021).

Besides marker selection, another challenge in DNA metabarcoding is quantitative analysis. Some factors can bias the number of read copies obtained for each individual or species, such as the number of mitochondria per cell, different‐sized individuals in the same sample, and amplification biases (Carvalho, 2022; Fonseca, 2018). Nonetheless, some studies have shown a positive correlation between the number of eggs or larvae in mock samples and the number of reads obtained for each taxon using DNA metabarcoding with an amplification step (Duke & Burton, 2020; Nobile et al., 2019).

This study used DNA metabarcoding to analyze the composition of ichthyoplankton sampled at the Neotropical megadiverse São Francisco River Basin, in Brazil. Additionally, the sensibility, specificity, and taxonomic resolution of two 12S markers were tested and compared with the traditional COI fragment used for DNA barcoding. The results obtained here will contribute to an improved method for ecological studies focusing on the ichthyofauna reproductive dynamics, and to design management and conservation strategies for the maintenance of fish reproduction locally.

## 2 MATERIALS AND METHODS

### 2.1 Sample collection

The São Francisco River Basin harbors at least 205 fish species (Barbosa et al., 2017), making it an excellent challenge for testing the sensibility and resolution of different markers within such complex Neotropical ichthyofauna. Fish collection was permitted by governmental agency ICMBio (license 70282‐4) and the study was approved by the institutional ethics committee (protocol number 27‐2019). Thirty ichthyoplankton samples (22 composed of eggs and eight composed of larvae) were sampled from the upper section of the São Francisco River and were named SF01 to SF30. The larvae samples were assembled using larvae of similar size (approximately 1 millimeter in average) to minimize bias due to biomass difference between species and relative read abundance estimation of input DNA from each pool.

### 2.2 Genomic DNA extraction

Genomic DNA was extracted from samples containing a pool of fish eggs and larvae fixed in ethanol. To ensure the complete evaporation of the alcohol, initially the excess was removed through pipetting, and then the microtubes were kept open for 3 h at 55°C. We then added 600 μL of TNES buffer to each sample and ground the bulk with a plastic pestle until only minuscule tissue fragments were left. Next, 20 μL of proteinase K (20 mg/mL) was added to each microtube. The samples were kept at 55°C until complete tissue digestion. Finally, the genomic DNA was extracted using a low‐cost saline protocol adapted from (Aljanabi & Martinez, 1997).

The samples were quantified using a Qubit 4 Fluorometer (Thermo Fisher) with a 1x dsDNA HS Assay Kit (Thermo Fisher) to verify the success of the DNA extraction. The samples were then diluted to 100 ng/μL.

### 2.3 DNA amplification

The DNA was amplified via polymerase chain reaction (PCR). For the 12S rRNA gene, NeoFish (193 bp, Milan et al., 2020) and MiFish (185 bp, Miya et al., 2015) markers were amplified. Milan et al. (2020) showed that both these markers are great alternative for fish detection and identification by testing different species delimitations methods. A fragment of the COI gene was amplified using a cocktail of primers targeting the 650 bp COI fragment standardized for fish DNA barcoding (Ward et al., 2005). For each sample, all PCR replicates were amplified with the same index and pooled together for sequencing. These replicates were used to mitigate amplification biases that could affect species detection and RRA estimations. The PCR reaction solution had a final volume of 20 μL, containing: 8.34 μL of ultrapure water, 0.16 μL of BSA (100 μg/mL), 10 μL of AmpliTaq™ Gold 360 Master Mix (Thermo Fisher), 0.25 μL of each primer, and 1.0 μL of DNA template. An additional 1.0 μL of ultrapure water was added for the negative control samples instead of the DNA template. For the positive control sample, 1.0 μL of template DNA from a saltwater fish species, *Prionace glauca*, was added.

PCR conditions consisted of initial denaturation for 10 min at 95°C, followed by 35 cycles of denaturation for 1 min at 95°C, primers annealing for 30 s at 56°C (COI), 60°C (MiFish), or 63°C (NeoFish) and extension for 30 s at 72°C, with a final extension for 7 min at 72°C.

The PCR results were checked using 1.8% agarose gel electrophoresis. Both 12S markers presented bands of the expected size for all samples, including the positive control, whereas for COI, the sample SF08 failed to produce any bands but was also included in the sequencing step. The negative control samples did not produce any bands but were also sequenced with the other samples.

### 2.4 Library preparation and DNA sequencing

According to the manufacturer's protocol, one library for each marker was prepared using the Collibri™ PCR‐free Kit (Thermo Fisher). The libraries were quantified at the start of the preparation, after each major step, and at the end, by fluorometry. The 12S libraries were sequenced in a MiniSeq™ Sequencing System (Illumina) using a MiniSeq Mid Output Kit (300‐cycle), and the COI library was sequenced in a MiSeq™ Sequencing System (Illumina) using a MiSeq Reagent Kit v3 (600‐cycle).

### 2.5 Bioinformatic analyses

The bioinformatics analyses were carried out using a customized pipeline written in R v4.4.0 (R Core Team, 2021). Briefly, primer removal was performed using Cutadapt (Martin, 2011). The DADA2 package (Callahan et al., 2016) was used to filter reads with *Q* > 20, to merge read pairs into Amplicon Sequencing Variants (ASVs) using its quality‐aware algorithm to remove chimeric ASVs, and to perform taxonomic assignment against a custom 12S sequence reference database for MiFish and NeoFish markers. This custom database was derived from the 12S library developed by Milan et al. (2020) for 12S markers containing 261 DNA sequences of 129 Neotropical fish species. The taxonomic assignment for the COI marker was performed using local BLASTn (Camacho et al., 2009) against the NCBI nucleotide database (Sayers et al., 2023; NCBInt). Percentual identity thresholds were applied for identifications at the species level for COI (98%) and 12S (99%). The RRA (relative read abundance) was determined by dividing the absolute counts of each ASV by the sum of the absolute counts of all ASVs in a sample.

To compare species identifications between markers, Venn Diagrams were built using the web application Lucidchart (https://lucid.app/). A Permutational Multivariate Analysis of Variance (PERMANOVA) was performed to calculate the dissimilarities between the qualitative and quantitative diversity of fish taxa detected by each marker using the taxon presence/absence‐based Jaccard dissimilarity index and the proportional read abundance‐based Bray–Curtis dissimilarity index, respectively, with 1000 permutations. We then used a principal coordinate analysis (PCoA) to plot and visualize the differences between the markers in taxa composition. Calculation of the dissimilarity indexes and PCoA analysis were performed using the commands “adonis2” and “betadisper,” respectively (vegan 2.5‐2 R package). Correlation analyses were performed to test for similarities between the species identified by each marker, using the R packages ggpubr (Kassambara, 2022) and ggplot2 (Wickham, 2016). For this purpose, the relative read abundance (RRA) of each identification in each sample was plotted, using one marker per axis. The dispersions were used for determination of correlations, with coefficients and significance values, using the lm method.

Due to the maximum 600 bp length limitation of the sequencing technology available, the forward R1 and reverse R2 COI reads could not be merged by overlap to reconstruct the barcoding amplicon, as each strand covers a different region of the COI gene with possible distinct variations for each taxon. Therefore, reads R1 and R2 were analyzed separately, and each sample's taxonomic assignment results were combined.

The ASVs found in the negative controls were removed from all other samples. Additionally, considering that the high throughput could amplify contaminations not detected by negative controls, and the risk of false positives, but also aiming not to exclude underrepresented taxa, only ASVs with more than 0.01% of relative read abundance (RRA) in each sample were considered.

## 3 RESULTS

All primer sets produced successful sequencing results for most samples. However, one sample (SF08) for COI did not produce any amplification, even after further DNA purification, quantification, and a new PCR adjustment. Nevertheless, SF08 was successfully amplified and sequenced using the 12S markers Mifish and Neofish. A low number of reads were observed for SF04 and SF08, resulting in only one and three reads, respectively, despite the latter not presenting any problems in the amplification process, resulting in 93.33% (two failed samples out of 30) sequencing success. Notably, both 12S markers resulted in 100% amplification and sequencing success, with at least 38,697 reads in a sample (SF27) for MiFish, and 37,228 reads (SF10) for NeoFish.

The sequencing effort resulted in 4,505,309 reads for all markers after quality filtering. The number of reads showed considerable differences among markers and samples. COI produced 584,309 reads, averaging 19,477 reads per sample, ranging from one (SF04) to 29,485 (SF11). MiFish presented 1,919,545 total reads, averaging 63,985, with a minimum of 38,697 (SF27) and maximum of 83,734 (SF23). Sequencing with NeoFish resulted in 2,001,455 reads, with an average number of 66,715 per sample, varying from 37,228 (SF10) to 96,285 (SF23). After BLASTn searches, 1699 COI reads remained without taxonomic assignment, and 69 were assigned to Bacteria. On the other hand, all MiFish and NeoFish reads were assigned to fish taxa.

**FIGURE 1:**
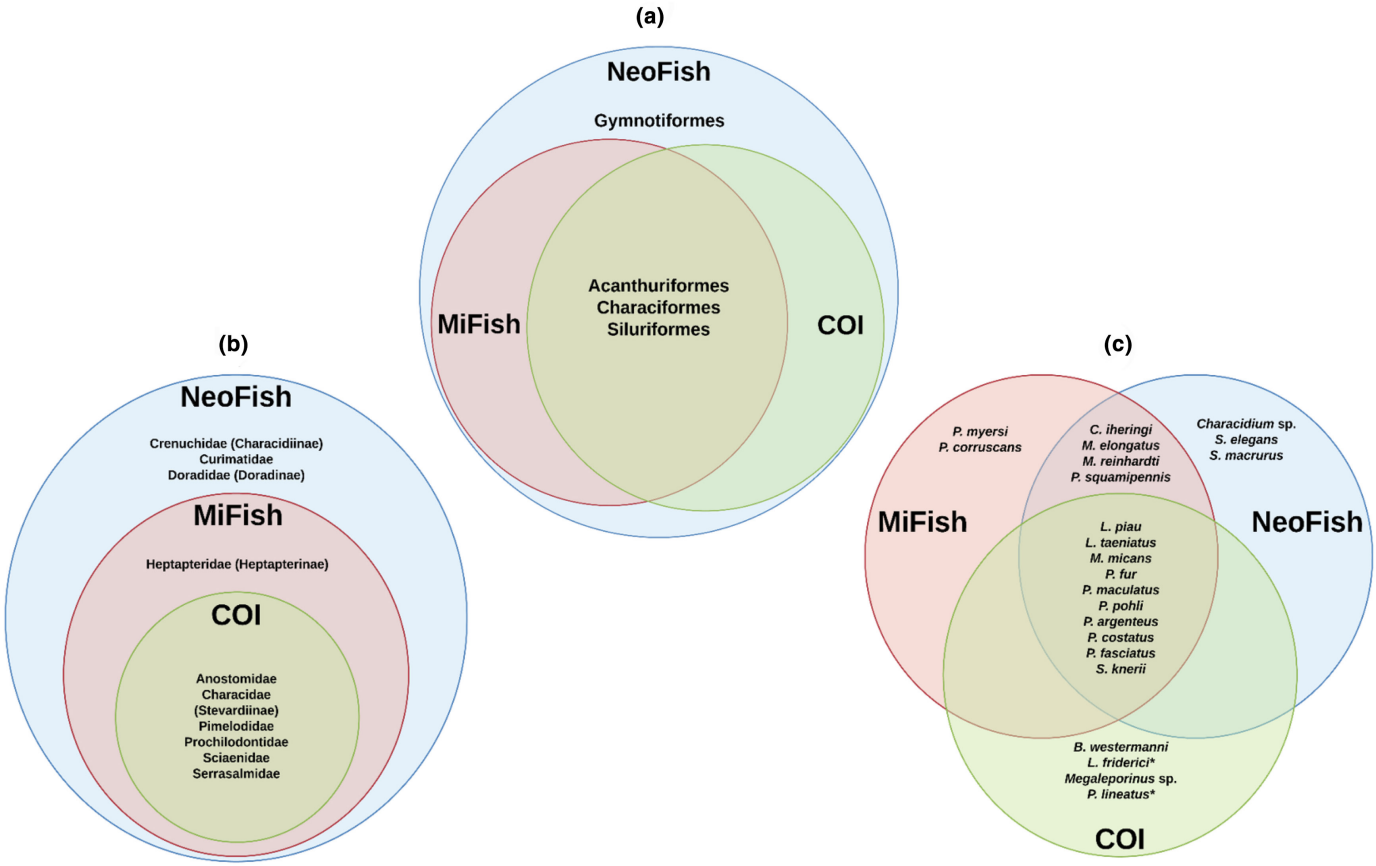
Venn diagrams recovered by each molecular marker MiFish, NeoFish, and COI considering distinct taxonomic levels: (a) order, (b) family/(subfamily), and (c) genus/species. Species marked with an asterisk (*) have not been reported for the São Francisco River Basin.

**FIGURE 2:**
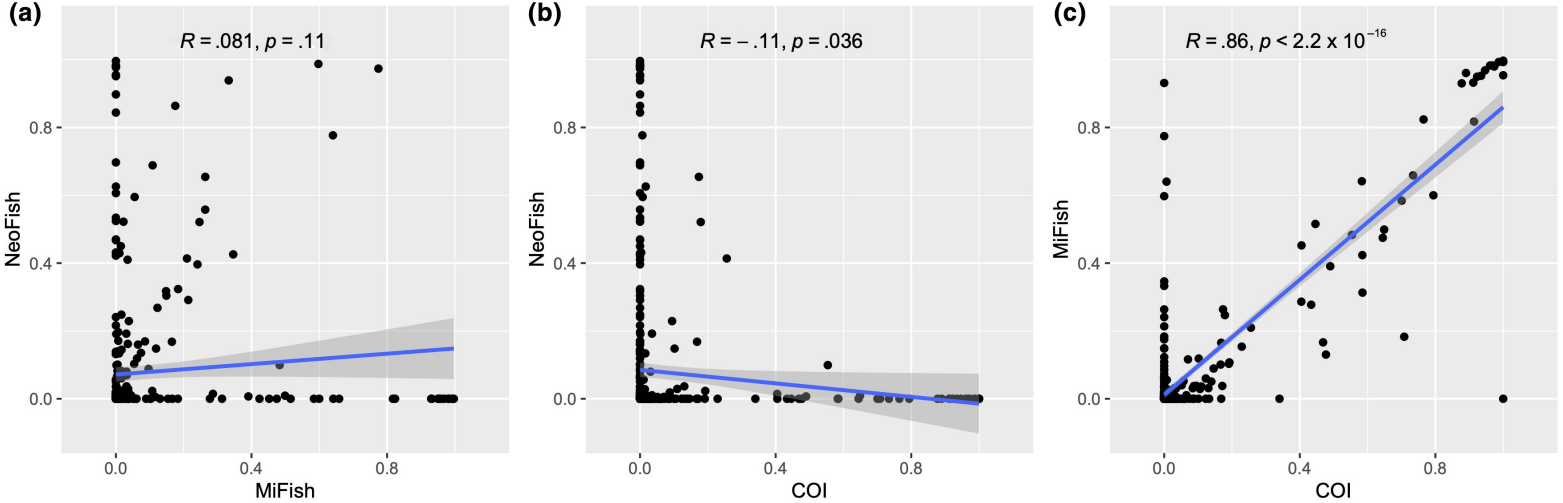
Scatter plots correlation between each molecular marker and the identified species in all ichthyoplankton samples. Each dot represents an identification in a sample, plotted using its respective abundance in the sample as measured by the marker on the corresponding axes. Blue lines show the regression lines with the confidence intervals in dark gray. *R* and *p* values are displayed for each correlation.

ASVs were assigned to 26 fish taxa, from which 22 were identified at the species level, two at the genus level, and three at the subfamily level. The 12S marker NeoFish was able to detect the highest number of orders, families, genera, and the same number of species as MiFish. In contrast, COI detected fewer species, genera, and families than the other markers and the same number of orders as MiFish (Table S1, Figures 1, S1). The dispersion of relative read abundance values for each identification, plotted for each molecular marker pair (NeoFish vs. MiFish, NeoFish vs. COI, and MiFish vs. COI), shows that there is no correlation between the NeoFish and MiFish markers (*R* = .081, *p* = .11), nor between the NeoFish and COI markers (*R* = −.11, *p* = .036; Figure 2). However, a significant positive correlation was observed between the MiFish and COI markers (with *R* = .86, *p* < 2.2^−16^).

**FIGURE 3:**
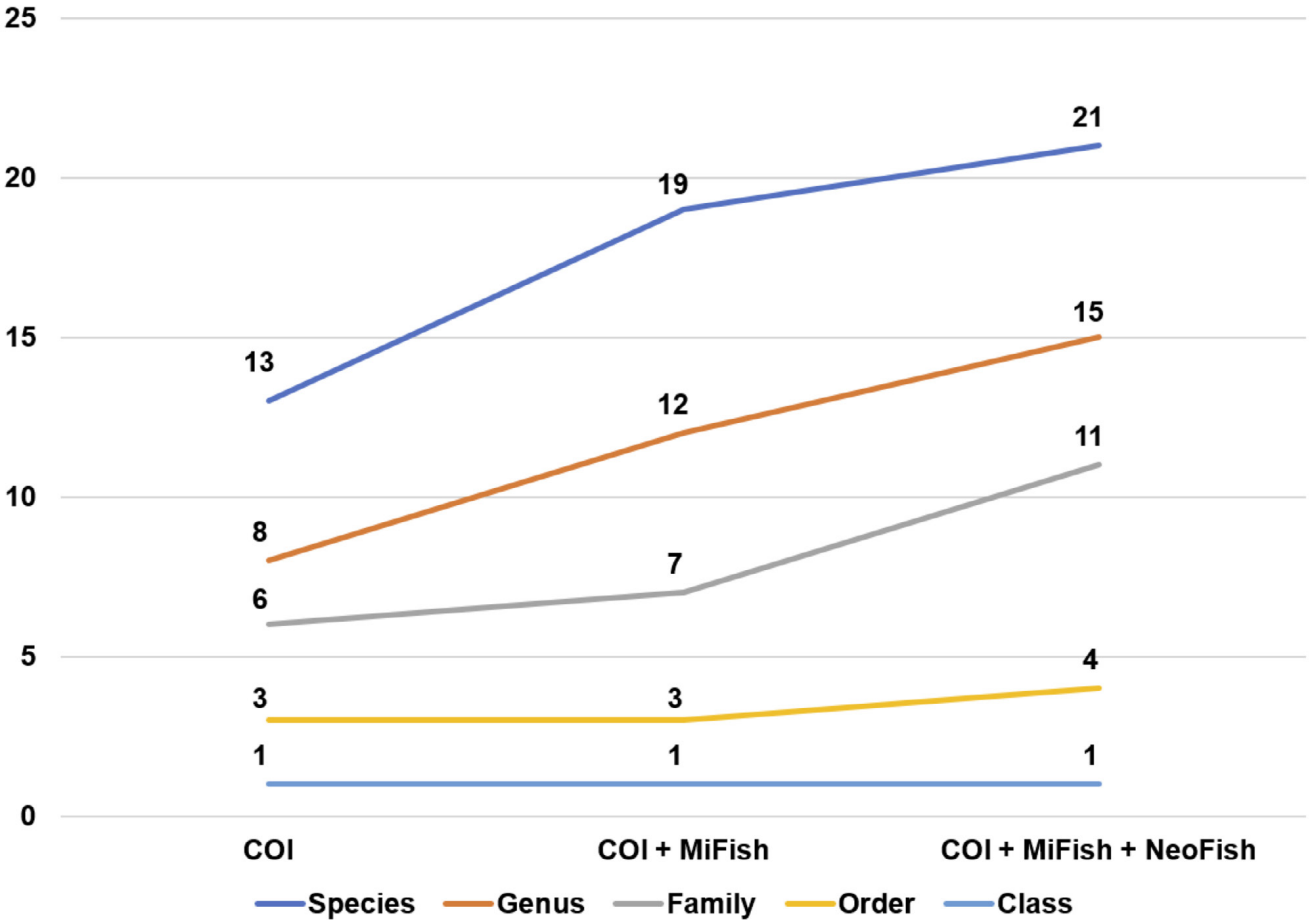
Improvement in detected taxa when using a single marker for the COI gene, combining COI and Mifish and combining COI, Mifish, and Neofish.

The combined use of the three markers increased the genera detection rates by 25%–87.5% when considering an initial analysis with only NeoFish or COI, respectively (Figure 3). The improvement in species recovery rates with the use of all three markers combined ranged from 31.25% to 61.54% when considering an initial analysis with either 12S gene markers or COI, respectively (Figure 3).

**FIGURE 4:**
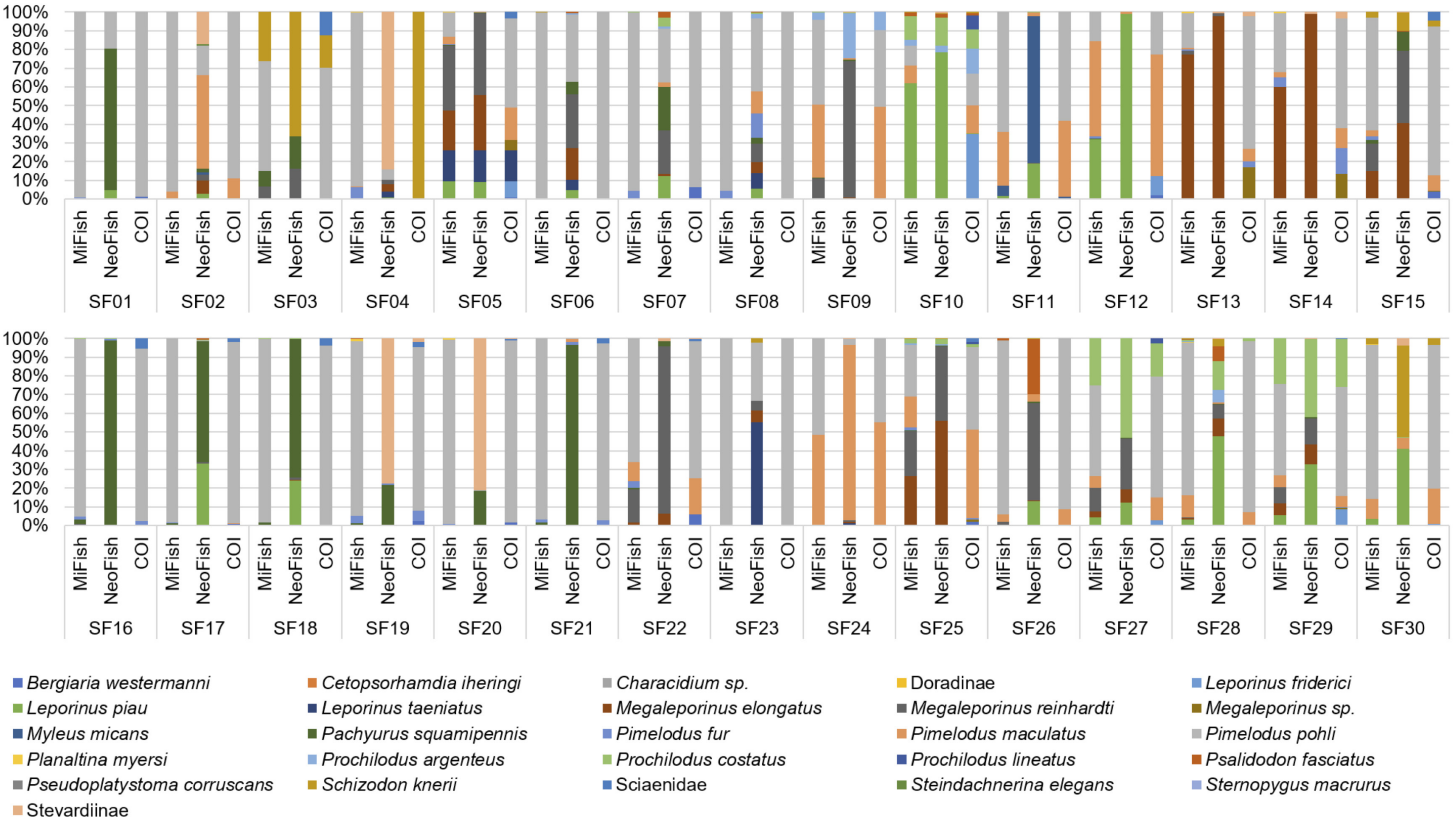
Relative read abundance (RRA) for each taxon, in each sample, with each marker.

The COI marker detected 16 taxa belonging to 13 species, eight genera, six families, three orders, and one class (Figure S1). Besides the 13 taxa identified at the species level, one was identified at the genus level, one at the subfamily level and another at the family level (Table S1). Among the 13 species, three were detected exclusively by the COI gene (*Bergiaria westermanni*, *Leporinus friderici*, and *Prochilodus lineatus*). The species *B*. *westermanni* was present in 12 samples, with an average RRA of 2.29%, ranging from 0.13% to 6.16%. The anostomid *L*. *friderici* was detected in eight samples, with the RRA ranging from 0.13% to 32.64%, and an average of 7.81%. Lastly, *P*. *lineatus* was present in three samples and had an average RRA of 3.60%, ranging between 1.20% and 7.12% (Figure 4).

With the 12S gene markers, 21 taxa were detected, including 18 species, 14 genera, 11 families, four orders, and one class (Table S1). One of these taxa was identified at the genus level (*Characidium* sp.) and two at the subfamily level (Doradinae and Stevardiinae). Of the 18 identifications at species level, eight were exclusively detected with the 12S gene, all indigenous to the São Francisco Basin: *Cetopsorhamdia iheringi*, *Megaleporinus elongatus*, *M*. *reinhardti*, *Pachyurus squamipennis*, *Planaltina myersi*, *Pseudoplatystoma corruscans*, *Steindachnerina elegans*, and *Sternopygus macrurus* (Figure 1, Table S1). Four of the eight species detected exclusively with the 12S markers were detected by both markers, but with some variation in samples and abundance. For example, the species *C*. *iheringi* was detected by MiFish and NeoFish in sample SF15, with 0.07% and 0.02% RRA, respectively (Figure 4).

Overall, RRA and taxon detection was not consistent between each marker (Figure 4). For instance, the most abundant taxon *P*. *pohli* had a total of 1.348.589 (70.79% of the total) for MiFish and 424.499 (73.02%) reads recovered for Neofish. Notably, the highest RRA detected for NeoFish was *M*. *elongatus*, with 236.332 (20.90% of the total) reads. Additionally, in some samples (e.g., SF05, SF13, SF14 and SF15) where both MiFish and COI detected multiple taxa for Pimelodidae, NeoFish was not able to identify any taxa for this family (Figure 3).

**FIGURE 5:**
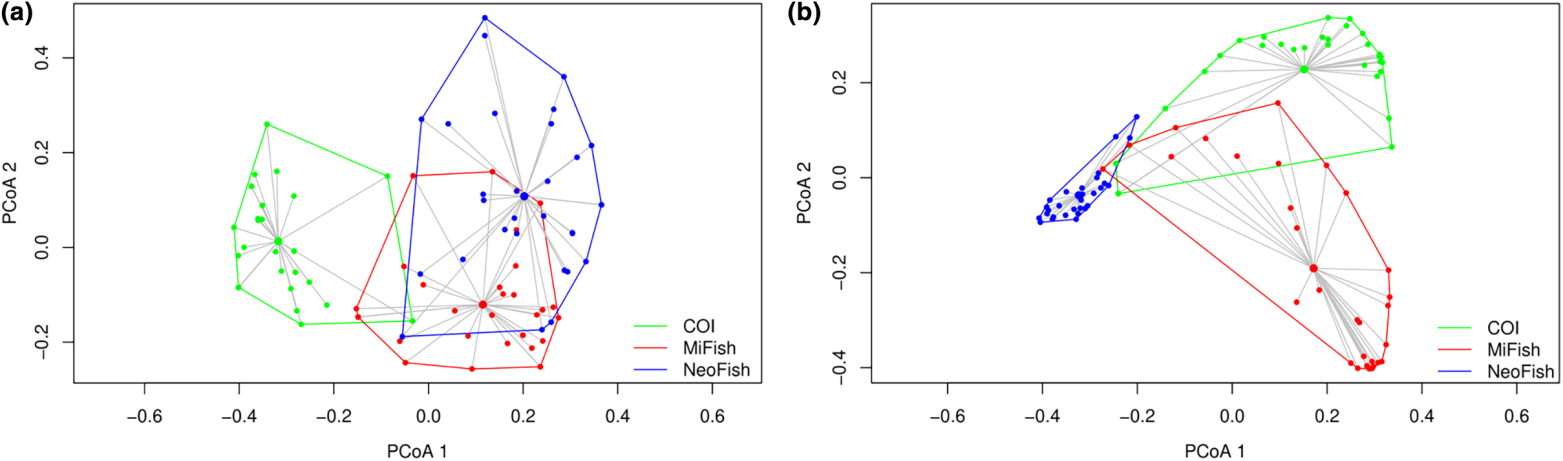
PCoA plots of 30 ichthyoplankton fish communities detected using three different molecular markers: COI, Mifish, and Neofish. Analyses were conducted using (a) Jaccard coefficient considering the presence/absence of fish tax and (b) the Bray‐Curtis coefficient, which uses a matrix of abundance based on each taxon.

The PERMANOVA evidenced significant differentiation of fish communities using distinct molecular markers (Table 1). The influence of marker choice on taxa recovered in each sample was significant for both presence‐absence (Jaccard) and RRA (Bray‐Curtis) analyses (Table 1). While there is considerable overlap between both 12S markers considering only presence‐absence of each taxon in each sample, as can be seen in the PcoA plot (Figure 5a), the analysis taking taxa abundance into account revealed a slight overlap between MiFish and COI (Figure 5b).

**TABLE 1:** Summary of PERMANOVA results (*R* ^2^‐effect sizes and significance level) showing the effect of marker choice on taxa recovered. Df, Degree of freedom; Sum of Squares, Value of Test *F*; and *p*‐value associated with the *F* score.

Index	Df	SumSqs	*R* ^2^	*F*	*p*
Jaccard	2	6.050	.1881	10.079	.001
Bray‐Curtis	2	7.898	.2283	12.869	.001

## 4 DISCUSSION

DNA metabarcoding has become an essential tool for species inventory and monitoring. However, its use in identifying ichthyoplankton is still incipient in the Neotropics, with several methodological challenges and biases still needing to be tackled (Carvalho, 2022). Considering the crescent demand for innovative techniques to unravel the complex reproductive dynamics of fish communities for both research and practical applications, there is an underlying need for the continuous refinement of this methodology. The COI gene has been commonly used as the marker of choice (Mariac et al., 2018; Nobile et al., 2019) because of its well‐established primers and complete reference sequences libraries encouraged by the global initiative Fish Barcode of Life. Here, each molecular marker recovers a distinct community structure when considering both quantitative and qualitative analysis.

Using a marker of choice still raises concerns since using several markers is still expensive when using HTS and because each marker has distinct amplification biases and taxonomic resolution (Deagle et al., 2014). The high interspecific variability of the COI gene, when compared to other mitochondrial genes (Hebert et al., 2003), can help differentiate closely related species. However, the same high variability creates the need to use universal degenerate primers with lower specificity than those designed for more conserved genes. Also, it hinders the design of internal minibarcodes for COI (Deagle et al., 2014). In the present study, while COI presented 93.33% sequencing success and some of its sequences remained unassigned or were assigned to Bacteria, both 12S markers were successfully sequenced for all samples, and all their sequences were assigned to fish taxa. Additionally, the technology used for sequencing limits the total fragment size to 600 bp, precluding the merging of both COI strands from forming the full‐sized barcode; therefore, each strand was analyzed independently. The loss of resolution power caused by this could explain why COI detected fewer species, genera, and families than the 12S markers and why two of the three exclusive species level identifications were assigned to nonnative fishes closely related to species from São Francisco.

Minibarcode markers for the 12S gene have been developed and applied to environmental DNA metabarcoding studies (Milan et al., 2020; Miya et al., 2020; Sales et al., 2021) and, more recently, to ichthyoplankton studies as well (Jiang et al., 2022; Van Nynatten et al., 2023). One of the main concerns when using these markers is the conserved nature of the gene, which can impact their ability to differentiate closely related species, especially in diverse regions. However, the current study shows that both MiFish and NeoFish were able to successfully identify and distinguish multiple congeneric species, such as *Leporinus piau* and *L*. *taeniatus*, *Megaleporinus elongatus* and *M*. *reinhardti*, *Pimelodus fur*, *P*. *maculatus* and *P*. *pohli*, and *Prochilodus argenteus* and *P*. *costatus*. Moreover, the 12S markers have a higher species detection sensibility than COI, considering that the exclusive fishes they retrieved were underrepresented, with low RRA. This could result from low‐efficiency primer binding by COI, which can lead to a lack of amplification (Zhang et al., 2020). Moreover, the relatively low number of overall taxa detected (*n* = 26) in comparison with the described regional biodiversity (*n* = 205) is likely not related to the detection power of the DNA metabarcoding method, but rather due to the sampling region, since we sampled only the Upper São Francisco River, combined with the selectiveness of the sampling method that targets only drifting ichthyoplankton.

Database completeness is another variable that directly impacts species detection, as a lack of reference sequences for a given species may hamper accurate taxonomic assignment (Collins et al., 2019). This aspect has affected both COI and 12S markers in this study. For instance, while *Pachyurus squamipennis* is not represented by any COI reference sequence in the public databases and was exclusively detected by 12S, the only native species retrieved solely by COI, *Bergiaria westermanni*, does not have any 12S representative sequence in neither the public nor our custom library. These limitations highlight the importance of continuous sequencing efforts to broaden reference sequence databases, especially for megadiverse regions.

Considering that each marker has advantages and limitations, some studies suggest combining multiple primer sets to increase taxonomic coverage (Liu & Zhang, 2021; Zhang et al., 2020). In a metabarcoding study using multiplexed markers to identify zooplankton mock communities, Zhang et al. (2018) demonstrated that a multi‐maker approach can improve species detection and allow the cross‐validation of taxa detected by each marker. Our results support this conclusion, as using the three markers combined increased the genera detection by up to 87.5% and species detection by up to 61.54%. Therefore, employing multiple markers, whenever feasible, reduces the likelihood of overlooking species or incorrectly classifying them due to the absence or mislabeling of sequences in the reference database (Locatelli et al., 2020).

Discrepancies between markers were observed in the quantitative analysis using the RRA values. Although some studies with mock samples of eggs (Duke & Burton, 2020) and larvae (Nobile et al., 2019) yielded a positive correlation between input organisms and output reads for each species, the results from this study support the idea that amplification bias is one of the main pitfalls for quantitative metabarcoding analyses, as already reported elsewhere (Carvalho, 2022; Fonseca, 2018). Moreover, while the correlation analyses showed positive correlation between the RRA values found for MiFish and COI (*R* = .86, *p* < .01), low‐efficiency primer binding to Siluriformes and especially Pimelodidae sequences may have led to different abundance patterns for NeoFish when compared to the other two markers. However, despite the significant positive correlation between MiFish and COI, many identified species were detected by only one of these markers (Figure 2). The inefficiency of the Neofish marker to amplify Siluriformes has already been reported using DNA metabarcoding of Neotropical fish mock communities (Hilário et al., 2023). Therefore, such biases in the amplification and detection of species by only one marker are in agreement with our results using real communities and reinforce the importance of using multiple markers when conducting large‐scale monitoring programs using DNA metabarcoding analysis.

In conclusion, using multiple markers from two distinct genes and lengths allowed an increased taxonomic coverage and robust taxonomic classification of complex Neotropical ichthyoplankton communities. Finally, precaution is still needed when inferring species abundance based on DNA metabarcoding data when using PCR‐dependent protocols since it is marker dependent. Nonetheless, ichthyoplankton metabarcoding offers superior resolution and feasible scalability compared to traditional techniques, and provides qualitative information, which is paramount for characterizing reproducing species and definition of conservation strategies.

## AUTHOR CONTRIBUTIONS

**Daniel Fonseca Teixeira:** Data curation (equal); formal analysis (equal); investigation (equal); methodology (equal); resources (equal); software (equal); validation (equal); visualization (equal); writing – original draft (equal). **Heron Oliveira Hilrio:** Data curation (equal); formal analysis (equal); methodology (equal); software (equal); validation (equal); visualization (equal). **Gilmar Bastos Santos:** Funding acquisition (equal); project administration (equal); writing – review and editing (supporting). **Daniel Cardoso Carvalho:** Conceptualization (equal); funding acquisition (equal); investigation (equal); project administration (equal); resources (equal); supervision (equal); validation (equal); writing – original draft (equal); writing – review and editing (equal).

## Supporting information


Appendix S1.

## Data Availability

Raw Illumina sequences and corresponding metadata are deposited in the Zenodo repository (https://doi.org/10.5281/zenodo.8038835).
