# Supplementary material for: DNA metabarcoding assessment of Neotropical ichthyoplankton communities is marker‐dependent
Source: Ecol Evol. 2023 Oct 20;13(10):e10649. doi: 10.1002/ece3.10649 (PMC10587807; doi:10.1002/ece3.10649)
Supplement: Supplementary file 1 — Appendix S1. [file ECE3-13-e10649-s001.docx]

Title: Qualitative and quantitative DNA metabarcoding is marker-dependent for assessment of Neotropical ichthyoplankton communities

Journal: Molecular Ecology Resources

Authors: Daniel Fonseca Teixeira^1, 2^, Heron Oliveira Hilário^1^, Gilmar Bastos Santos^1^, Daniel Cardoso Carvalho^1*^

Affiliations:

1- Post-Graduate Program in Vertebrate Biology, Pontifical Catholic University of Minas Gerais, PUC Minas, Belo Horizonte 30535-610, Brazil; carvalho.lgc@gmail.com (D.C.C.); heronoh@gmail.com (H.O.H); gilmarsantos4@hotmail.com (G.B.S.)

2- Post-Graduate Program in Genetics, Federal University of Minas Gerais, Belo Horizonte 31270-901, Brazil; danielfonsecat@gmail.com (D.F.T.);

*Corresponding author: carvalho.lgc@gmail.com


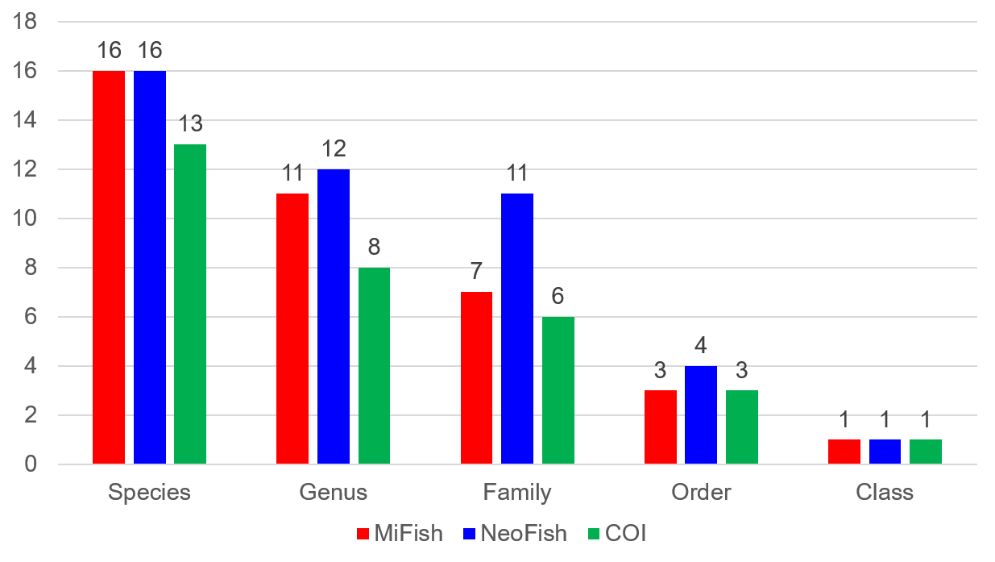


Figure S1. Number of species, genera, families, order, and classes detected by each marker.

Table S1. Twenty-six taxa were detected within all samples by the three markers Mifish, Neofish and COI. An asterisk (*) highlights the species not reported for the São Francisco River Basin.

| Order | Family | Subfamily | Genus/Species | MiFish | NeoFish | COI |
| --- | --- | --- | --- | --- | --- | --- |
| Acanthuriformes | Sciaenidae | - | - | - | - | X |
|  |  |  | *Pachyurus squamipennis* | X | X | - |
| Characiformes | Anostomidae | - | *Leporinus friderici** | - | - | X |
|  |  |  | *Leporinus piau* | X | X | X |
|  |  |  | *Leporinus taeniatus* | X | X | X |
|  |  |  | *Megaleporinus elongatus* | X | X | - |
|  |  |  | *Megaleporinus reinhardti* | X | X | - |
|  |  |  | *Megaleporinus* sp. | - | - | X |
|  |  |  | *Schizodon knerii* | X | X | X |
|  | Characidae | Stethaprioninae | *Psalidodon fasciatus* | X | X | X |
|  |  | Stevardiinae | *-* | X | X | X |
|  |  |  | *Planaltina myersi* | X | - | - |
|  | Crenuchidae | Characidiinae | *Characidium* sp. | - | X | - |
|  | Curimatidae | - | *Steindachnerina elegans* | - | X | - |
|  | Prochilodontidae | - | *Prochilodus argenteus* | X | X | X |
|  |  |  | *Prochilodus costatus* | X | X | X |
|  |  |  | *Prochilodus lineatus** | - | - | X |
|  | Serrasalmidae | Serrasalminae | *Myleus micans* | X | X | X |
| Gymnotiformes | Sternopygidae | Sternopyginae | *Sternopygus macrurus* | - | X | - |
| Siluriformes | Doradidae | Doradinae | *-* | - | X | - |
|  | Heptapteridae | Heptapterinae | *Cetopsorhamdia iheringi* | X | X | - |
|  | Pimelodidae | - | *Bergiaria westermanni* | - | - | X |
|  |  |  | *Pimelodus fur* | X | X | X |
|  |  |  | *Pimelodus maculatus* | X | X | X |
|  |  |  | *Pimelodus pohli* | X | X | X |
|  |  |  | *Pseudoplatystoma corruscans* | X | - | - |
